# Medical Jurisprudence.—Insanity in Relation to Crimes*1. “Unsoundness of Mind in Relation to Criminal Acts;” an Essay, to which the first Sugden Prize was this year awarded by the King and Queen’s College of Physicians in Ireland; by John Charles Bucknill, M. D., London, &c., &c., and Physician to the Devon County Lunatic Asylum. London, Samuel Highly, 82 Fleet Street. 1854.

**Published:** 1856-06

**Authors:** 


### SELECTIONS.

### *Medical Jurisprudence.—Insanity in relation to Crimes.**

The legal relations of crime and disease continue to engage the earnest attention of men distinguished in jurisprudence and medicine. The common law of England, which, on this subject, is elementally the law of most of the States of our Union, has been gradually attempered, by the influence of advancing science, and by a readier judgment of experts, to a more genial conformity with the requirements of ripening civilization. What the common law lacks, from apparent want of plasticity, to meet the actual or presumed necessities of the subject, is in a measure supplied by statutory provisions, by the decisions of judges warping the law to particular cases, or by, what is practically more efficacious, the verdicts of juries.

Still, it is a grave question both in England and this country, whether the existing state of the law respecting mental unsoundness in connection with acts ordinarily criminal, is as satisfactory as an enlightened and discriminative legislation can make it. As an expressive evidence of the interest manifested in this matter abroad, it may be observed, that the essay of Dr. Bucknill, the title of which we have given as partly suggestive of this article, is the offspring of a prize, instituted by no less a personage than a late Lord High Chancellor of England (formerly eminent as Sir Edward Sugden,) for the best essay on "Insanity, its Responsibility and its Negation, and the Relation between the two."

These topics are treated by the essayist with boldness, and, so far as regards the legal relations of insanity, in a conservative spirit that must almost satisfy a lawyer; which is saying a good deal, when we bear in mind the alleged tendency of the medical profession to refine away the legal principles which have so long been maintained, with more or less strictness, as the standard of the relations between crime and disease.

The "Treatise on Medical Jurisprudence," by Mr. Wharton and Dr. Stillé, is a work of no ordinary merit, particularly in those parts devoted to the discussion of the purely legal relations of insanity. It is not more meritorious for the research and comprehensiveness which it displays, than for its order and arrangement, and the excellence of its analytical table. Its singular feature is the association of a jurisprudent

---

* 1. "Unsoundness of Mind in Relation to Criminal Acts;" an Essay, to which the first Sugden Prize was this year awarded by the King and Queen's College of Physicians in Ireland; by John Charles Bucknill, M. D., London, &c., &c., and Physician to the Devon County Lunatic Asylum. London, Samuel Highly, 32 Fleet Street. 1854.

2. "A Treatise on Medical Jurisprudence," by Francis Wharton and Moreton Stille, M. D. Philadelphia: Kay & Brother. 1855.

and a physician to produce a work designed to be of equal authority to two professions which are not apt to concur in their opinions on mixed questions, for reasons which we shall presently suggest.

Law, being a *general* rule of action, is, of necessity, not plastic enough to be moulded to all possible circumstances and conditions. In this respect it falls short of equity jurisprudence; which, in its turn, does not reach questions of felony or other high crimes. Neither does law profess to be ethical, but leaves ethics to conscience and to God. It cuts a rigorous, inflexible, and sharp line on either side of which the points most adjacent, although sundered, are so contiguous as to be often confounded. In truth, it sometimes, from its spirit of generalization, severs justice and right in the very middle. As Dr. Bucknill expresses, "It is a Rubicon, on one side of which Cæsar is a servant of the state; on the other, a traitor and a rebel." Some venerable legal maxims betray this characteristic with great force. Thus, "Ignorance of the law excuses no man;" yet much of the knowledge of it is professional and demands the devotion of a lifetime; and therefore it would seem that some degrees of ignorance should be excusable. So, "If one knows the difference between right and wrong, he is responsible for the wrong;" yet, with all his *knowledge*, he may physically or mentally lack the *power* of shunning the wrong or doing the right; and therefore to the eye of God appear inculpable, although no human eye can detect the weakness which is his real apology, and is morally his defence.

In a rough way, however, and for the common purposes of civil government, such comprehensive and absolute maxims are true and useful. They cover the great mass of realities submitted to human arbitrament, although they fail of precise adaptability to possible, and even frequent, chances. At the best, mankind can be governed and restrained only in a rough way, by any human codes; as they can be treated for diseases only in a rough way by medical skill. Yet time, experience, and circumstances, have effected substantial modifications in various branches of the law, as well as medical practice; and, doubtless, other branches of both will undergo similar modifications, as exigences demand. Lord Mansfield was a great judge; and his greatness consisted mainly in the bold perspicacity that enabled him to adapt the common law to the altered circumstances of society, without impairing its foundations, or the force of its legitimate precedents. Under his decisions, commercial law, for instance, assumed a practical applicability to the new demands of commerce and trade, by a simple expansion and development of established principles, without any violent deflection from their true spirit and intent. An enlightened and bold mind will educe from the common law a conformity to the shifting phases of life and business, which a narrow and weak one will never discover or apply. Its great merit is that every well-established principle and fact of science and art becomes a part of it, as much as Christianity and the almanac, which have been decided to be so.

The criminal law of England always owed much of its severity, as well as of its confusion of offences and penalties, to parliamentary acts. Generally, the real spirit of the common law was less harsh and more uniform. The statutes made capital crimes out of many acts comparatively trivial, and visited them with punishments as severe as any inflicted by the common law of the land on the most flagrant violations of human rights. Thus, minor offences, of the grade of misdemeanors by the common law, were magnified by the statutes into grave felonies that involved transportation for life, or hanging, as their penalty. A forgery became as heinous as a murder; a violation of property, as a violation of the person or the life. No worse punishment could be inflicted on the most reckless homicide than was the doom of Dr. Dodd for counterfeiting the signature of Lord Chesterfield to a bond; "a crime," according to Dr. Johnson, which, "morally or religiously considered, has no very deep dye of turpitude. It corrupted no man's principles; it attacked no man's life. It involved only a temporary and reparable injury." And yet it incurred, by the statutes, the penalty reserved by the common law for the most deliberate and cruel murder.

Of lunatics and idiots the common law of England was always considerate. It discriminated according to its lights, respecting the *responsibility* of alleged criminals for their acts; and although, in compliance with its imperturable maxim, it "allowed ignorance of the law to excuse no man," it did excuse every man who wanted the knowledge of right and wrong, by reason of congenital defects, or of disease, impairing his faculties. Common-law principles were humane in their spirit, but they were not always well defined; and they were sometimes, as they are still, misapplied or perverted. It is a part of the purpose of Dr. Bucknill's essay to show that, even at this day, the English courts are clouded by obscure and conflicting definitions of the law regarding insanity, as laid down by the highest authorities; and to suggest modifications of it with respect to forms of proceeding, to judicial discretion, and to the modes of restraint and correction.

"Stand on decisions," is the maxim of the judge, which implies a tendency to generalize, and reduces to a common standard: "Test each case by its symptoms," is the maxim of the physician, which implies a tendency to individualize, and decide every case on its own peculiarities. That such diverse tendencies should produce a clashing between the professions is not singular; and when the doctors of law and the doctors of medicine criticise each other's definitions, judgments, and conclusions, on points involving the particular skill and knowledge of each, the difference in their intellectual training and their professional bent should always be allowed for.

Besides, what the law *is*, and what the law *ought to be*, are obviously very different questions; the one peculiarly within the province of legal experts, and the other, an open question for experts and inexperts of all professions.

Much of the difficulty in applying and modifying legal principles to cases of alleged crime, when unsoundness of mind is pleaded, arises from the obstinate fact that human laws can only take cognizance of acts, and of motives as developed by acts; they cannot rise to the subtleties of ethics or casuistry. But the Divine law takes cognizance of *motives* as well as of acts, and judges with equal severity of both. Whether, in human judgment, a man deserves reward or punishment, is always a mixed question: it respects both the intent and the deed. A human tribunal, on account of its infirmity, can no further judge of the intent than by inference from the deed, and from other external circumstance that illustrate the intent. The Divine tribunal, on the other hand, needs no overt act to betray a purpose: the purpose itself is no sooner conceived than it is known to the Omniscient eye. Hence, Divine justice can never be reproached with error; while error is the constant reproach of human tribunals. To quote Dr. Bucknill, "They do the best they can with imperfect knowledge, and look to *general good results*, rather than to an *unattainable exactness*."

Legally, lunacy, and unsoundness of mind, are equivalent or convertible terms. Unsoundness (insanity) is a term that covers every phase of mental infirmity, caused by disease, from a flaw to a perfect wreck. In the criminal aspect, lunacy is not simply what it was once, and is even now properly regarded to be—madness, fury, violence—whether continual or interrupted by lucid intervals or remissions: it is *every defect* of the mental faculties produced by *disease*. Idiocy is wholly irresponsible in this country as well as in England; but unsoundness of mind, under the constructions of *our* courts, is not a *complete* shield. It covers only the acts committed under the particular shadow of the mind; while all committed within the circle of its light are regarded as more or less culpable.

Sanity, however, is always legally presumed: it is the normal state of the mind; and insanity must be proved as a state of *disease* or the *result of it*. Moral insanity, as commonly understood and defined, does not fall within the precedents of the common law, and is not provided for by statute, unless it be under the general term, "insanity." It may be as palpable to the eye of Omniscience, and possibly to the scrutiny of an expert (*expers expertissimus*, he must be), as many forms of physical disease; but to legal tribunals it is shadowy and intangible: its very name of "moral insanity" seems to deprive it of legal recognition as a disease within the compass of exact definition and discrimination; and it is even doubtful whether it be a *disease;* and, therefore, if tolerated as a plea of irresponsibility, it would, like charity, cover the *multitude* of sins. Almost any man may satisfy his mind, if not his conscience,—a sane man, perhaps, the most readily,—that he has been surprised into a crime by some strange and irresistible impulse, some demoniacal instigation, some fatal propensity, or some unaccountable frenzy, that he could not master for its suddenness and its force. Such casualties may be, and doubtless are; but God only can judge of them. Human laws cannot: their nicest refinements are too gross for such subtleties. Besides, much of moral insanity, in the popular understanding of the term, is the want of discipline, and of habitual self-control; and nature, uneducated and unchecked, is, or very soon becomes, the spirit of Cain—a *propensity* to something wrong,—to theft, to perjury, to homicide. If such impulses, instigations, propensities, or frenzies are permitted to shield offence against punishment, St. Giles's and the Five Points might surfeit the courts with pleas of that character, the result, not of *disease*, but of *habit* not absolutely uncontrollable—of such defective discipline, and of such voluntary indulgence in vicious courses, as have deadened the moral sense, and confounded the appreciation, without obliterating the knowledge, of right and wrong, much less the power of choosing between them.

Here the lawyer and the physician come to a controversy, chiefly in consequence of the different tests which they are severally trained to apply. To those who are indifferent between the two, it may seem that the courts might as well be expected to hear and determine cases of casuistry, on the testimony of expert divines, as cases of moral insanity, on the testimony of medical experts. Both are worthy of competent tribunals, and both have ultimately the same most competent tribunal; and, happily for human infirmities, *God* is its *Judge*.

Insanity, in the contemplation of the law, is a *disease of the brain:* at all events, a *disease:* and this fact of *disease*, or the *result of disease*, is the *legal* touchstone of its validity as a defence against allegations of crime. None other can be safely or wisely allowed. Unless moral insanity, so called, can be satisfactorily proved to be a *disease*, or the *result of disease*, it is no legal shield against punishment, no mark of irresponsibility. As a mere plea, founded on subtleties, whether legal, medical, or psychological, and unsustained by proof of actual disease, it is not regarded by the law as of any moment whatever.

It is not to be disguised that, for some cause, *insanity* and *alibis* are the favorite defences in most cases of crime involving severity of punishment. Of the two, insanity is nowadays the more common. The *alibi* is somewhat musty and tattered, and only resorted to when either the actual truth, or determined perjury, will sustain it. Insanity (which may be regarded as an *alibi* of the mind) is treated, from humane motives, and most justly, with great consideration; and it is worth a reflection whether the presumed disposition of medical experts to spread its charitable mantle over idiosyncrasies, peculiarities, and defects which do not, in any ordinary legal sense, constitute lunacy, is not likely in the long run, wholly to disarm the unfortunate of their panoply, or at least to make it so penetrable to the shafts of ridicule as to be an uncertain defence.

Taking the common and the statute law, the often conflicting constructions put upon them by the courts, and the opinions of medical experts, together, there seems to be such a discordance and uncertainty as naturally to suggest the necessity of some legislative attempt to place the whole subject on a better footing. The man who, possessing a conservative spirit, and a mind reflecting truly the light both of law, of medicine, and of psychology, would frame a suitable code to define and establish the legal relations between unsoundness of mind and those acts, otherwise criminal, which spring from it, would not only be no common benefactor, but he would be much more than a common man. He should obviously be either expert himself, or in a position to command the expertness of others, both in law and medicine; and he should be free of the partisan taint of either profession. He would probably be forced to concede that *moral insanity*, until satisfactorily established as a disease, is too shadowy and undefinable to be dealt with, by human legislation, and by the ordinary tribunals, in regard to its criminal aspects; and "that its nature," in Dr. Bucknill's phrase, "is not thoroughly understood;" often difficult to distinguish (where it really differs) from mere perversion of the mind and affections caused by circumstances and habits that do not entitle it to complete exemption from legal responsibility. He would reflect, also, that society claims protection, as well as imbecile and unsound members, and often against them; and that the criminal code, as the chief part of its protection, should not be effeminated by a too diluted humanity or by too nice refinements. It must practically adjudicate particular cases by general rules, predicated upon the usual and ordinary modes and motives of human action; while to judge specially of each case by its peculiar symptoms and circumstances pertains to medicine, as respects bodily and mental disease, and to casuistry, as respects conscience. Yet medicine has its rough and general ways as well as law; for, according to Dr. Bucknill, "the drug and the dose are suited to the requirements of the patient [only] *as closely as medical knowledge can ascertain;* it is probable, however, that *too much or too liitle* is constantly given." In effect, then, the law seems to have this advantage; that if the dose prescribed by the *legal* dispensatory is too powerful for the disease, the humanity of juries, predominating over the strictness of their oaths, shapes the verdict so that the dose shall either not be administered at all, or shall be administered with suitable modifications. This is particularly the case with capital crimes.*

But, whatever refinement and adaptations the law may need or be capable of, it is certain that its true *intent* is the common intent of humanity—to allow to unsoundness of mind a perfect exemption from responsibility for all acts which fall within the shadow of that unsoundness, or are the natural offspring of it. The duty of the courts is to fulfill that intent; to confine juries to it; and, within the limits of their power and discretion, so to construe the law as that its humane purpose shall be effective. In the last particular they are, perhaps, somewhat straightened by precedents and judicial constructions, as well as by the obscurity of the subject. Such is Dr. Bucknill's opinion respecting the English tribunals, and such is probably the fact as to ours. The obvious benignity of our statutes, as well as of the common law, is somewhat chilled by glosses and constructions imbued with the spirit of the maxim "*stare decisis,*" of reverence for the antique and perverted expositions of the English law, and of jealousy respecting legislative interference with judicially established principles.

By the statutes of New York, a "*state of insanity*" exempts from punishment. The phrase seems to cover *any* unsoundness of mind, whether the particular act done has any connection with that particular unsoundness or not. It has been decided, however, by high legal authority, that the statutory phrase is not to be construed so broadly; but that the act of alleged criminality must be the distinct offspring of that unsoundness. Thus, if a monomaniac, insane as to the matter of theft only, should commit a homicide, unaccompanied by theft, or unconnected with such a design, his *unsoundness* would be no defence as to the homicide. The decision may be morally right, but it is questionable, perhaps, whether it conforms to the real intent of the statutes.

The exercise of any discretion, in criminal cases, for the tempering of justice to society with mercy to the accused, is legally confided to the executive authority, and not to the tribunals; subject to the practical qualification, that juries (as before intimated) sometimes leave on room for the exercise of any discretion but their own, by finding a verdict that absolves the accused. A humane jury will, in cases appealing to their sympathy, and showing what in France are called, "extenuating cir-

* In the State of New York, the statute defining *murder* places in the same category, as regards punishment, a homicide committed with the most *casual* (not to say merely *constructive*) premeditation, and a *deliberate, cold-blooded* murder, the supplement or auxiliary, perhaps, to some seduction or robbery. An additional clause might be framed to distinguish between *such* cases, and smooth the way of duty to juries.

cumstances," seize upon the most trifling evidence of insanity to justify a verdict in consonance with their sense of humanity, rather than with the rigidness of the law. Juries are usually above law, when law itself is not flexible enough to conform to the dictates of a reasonable sympathy; and then it is that their *legal* conscience surrenders to the dictates of their *moral* conscience, confident that the common suffrages of the humane will applaud their decision.

In an ethical view, responsibility may be perfect or imperfect. But its comparative imperfection cannot be exactly graduated by legal standards, although it may be approximately so, and perhaps as nearly so as legal standards can graduate anything. They may measure tons, or yards, or pounds, or inches, with sufficient accuracy for ordinary practical use; but they must, from their necessary generality, fail in the nicer and fractional degrees of weight and measure. They have no dernier scale or assay balance. "*De minimis non curat lex*" is a maxim highly expressive of the inadaptability of the law to the refinements of ethics, and even of equity. Both ethically and legally, a totally unclouded mind is sane, and therefore perfectly responsible. Ethically, a mind in shadow is imperfectly responsible, according to the extent of its eclipse. To define the perfect shadow of the eclipse is the problem which puzzles psychology and medicine, and much more the law. We do not expect the same heat from the sun, nor the same light from the moon, when obscured by intervening spheres, that we do when they show their uninterrupted splendor; but the one still gives heat, and the other light, appreciable, if not accurately measurable. So a man, whose mind is not wholly sound, may not be *wholly* guilty according to the extent of his capacity or derangement. The conditions of mental disease, on Dr. Bucknill's authority, must be allowed to modify responsibility *quantum valeant*. The *quantum valeant* is the stumbling-block to courts and juries, respecting which, however, they might make a much closer estimate than that of the English law; that seems to hold that *any* unsoundness of mind is legally equivalent to *total* unsoundness, and that a man who is not wholly responsible is therefore wholly irresponsible. An idiot knows nothing, or so little that it is counted as nothing; an insane person is not an idiot, but usually has faculties and perceptions of truth, more or less perfect, and, within their limited range, capable of control or restraint; and, unless furiously mad, he may, therefore, be held to a modified responsibility, and be a fit subject for a modified sort of correction; just as a child is, according to the degree of his intelligence and the development of his faculties. No one would assume that, because these are still immature and imperfect, they should be indulged with entire exemption from responsibility, although they do enjoy a degree of exemption proportioned to their ripeness and expansion. If restraint and punishment were spared to childhood, and reserved for years of maturity, what is bad in human nature would so overwhelm what is good, that nine cases in ten of adultness might claim at the hands of criminal justice the immunity demanded for *moral insanity;* to which, indeed, they would be next of kin, if not the very thing itself.

Unsoundness of mind assumes such various phases, and springs from such various causes (some of them habitual vices), that certain modes of correction and restraint, less severe than the discipline of prisons, and more rigid than that of asylums, might, perhaps, be wisely applied in cases of criminal propensity. In partial insanity, a well directed and thorough treatment of the sound faculties *might* overpower, subjugate, or at least balance, the unsound; for we know that a studious self-control enables many men to withstand and overcome propensities that seem irresistible, and even to contend against insanity itself. When *self-control* fails, the control of others is often a successful substitute. But such control, not being self-applied or inflicted, is *restraint;* in a modified way, it is *correction;* and correction is a milder term for punishment. Regarded as *restraint,* the welfare of society demands it; regarded as *correction,* the welfare of both society and the subject of the correction may justify it.

Medical experts differ, however, very widely on this point. It is maintained by some, that *any* degree of mental unsoundness should protect its victim from all forms of punishment, however graduated; and by others, that many manifestations of insanity are so dubious, faint, and obscure as to warrant the application of cautious punitive measures as correctives. A tender sympathy with human infirmity revolts at the idea of adding to the afflictions of the insane, in any mode, whether of restraint or correction; while a broad, and even a genial, humanity may venture to concede that correction, as well as restraint, though painful, may be serviceable.

However this may be, one thing is certain—that if the State undertake to make provision for idiots, lunatics and criminals, it should see that such provision is really adapted to its several purposes. Jails and prisons are mainly places of *punishment;* asylums, poor-houses, and hospitals are mainly places of *cure,* or of *charitable support.* Lunatics of criminal propensities, and who, but for their lunacy, would be convicted of crime, are fit subjects for neither of these, whether on their own account, or on account of those who must be their associates. An institution where due restraint, healthful labor, suitable correction, and professional care, may all be applied with discriminative reference to the case of each inmate, seems to be an obvious necessity, in all civilized states, in order to complete the circle of public charities. To enforce labor and inflict punishment on such whose case we are considering, as in a common prison, were inhuman: to allow them indiscriminately, the optional freedom from both, which an ordinary asylum permits, might be, to many, an unwise indulgence, and dangerous to all.

We are therefore disposed to urge somewhat zealously, as both Dr. Bucknill and Mr. Wharton do, the establishment of a separate institution for the confinement and special use of that class of lunatics who are alleged felons, and are either not tried, or not convicted, or not adjudged to the usual punishment, on account of insanity, or who are lunatics of criminal disposition; but who require a rigid isolation, and strict restraint of personal liberty, for the safety of society; and some of whom are presumably the proper subjects of corrective as well as curative measures. Such an institution should obviously be under the principal charge of an expert in insanity. If not as agreeable, it would certainly be as honorable and as useful a charge as the superintendency of an ordinary hospital or asylum. The principal should ostensibly be its medical adviser only, seeking the restoration to sanity of those committed to his keeping. The restraints and discipline demanded for the government and reform of the inmates should be administered by subordinates under his control or direction; and such provisions might be made as that those who are discovered, on some authorized investigation, to be really sane, but who have been judicially exempted from conviction and punishment, by erroneous or partisan verdicts of insanity at the time of committing the offence charged on them, should receive such severity of treatment as would not only conduce to their amendment, but atone for the fraud or error which saved them from the deserved judgment of the criminal law. That they cannot be exposed to a second trial for the offence of which they ought to have been convicted, is a good reason why the community should have the advantage which their false plea of insanity happens to give, and the protection of the restraint which it justifies. Simulated insanity cannot claim more favor than sanity; and when it is fairly caught, it should not complain of being roughly used.

The general supervision of our public charities is confided chiefly to legislative committees, or to officers of state, whose oversight is very indirect and formal. The multiplication of foundations for the support and care of idiots and lunatics demands a more efficient and careful supervision than this. There should be a permanent commission of lunacy and idiocy, composed of experts in medicine and law, the efficiency and experience of which should be secured by some order of rotation or reappointment, so that there should be always in service a suitable number of well-qualified men. One purpose of such a commssion should be to make a periodical, personal inspection of all jails, prisons, poor-houses, hospitals and asylums, and to report upon their condition and management; another purpose, to select from them such of the inmates as would be suitable subjects for the proposed institution for lunatics of criminal disposition, in case it should be founded. Such a commission, too, might be a useful auxiliary to the courts; for the members of it being experts, might be called upon either as *amici curiæ*, or as witnesses, to aid the determination of judges and juries, in questions of insanity. Whether experts should ever be witnesses, or only *amici curiæ*, or referees, is a question which at present we cannot investigate, although the whole subject of expertness (in its legal bearing) is interesting enough and important enough for a full discussion. In France, they are selected to take testimony and report to the tribunals, but are not examined as witnesses; and Dr. Bucknill suggests that they should be referred to by the criminal courts of England, as the Masters of the Trinity Company are referred to by the Admirality Courts, namely, as *amici curiæ*, and not as witnesses. But for the purpose of supervision alone, such a commission as we have suggested would be of sufficient value, if its objects were duly fulfilled, to justify its appointment and its expense.

The conclusions we reach are, that there is much in alleged, and probably in real unsoundness of mind that is not yet recognizable by the criminal courts, and which is beyond their legitimate capacity to deal with satisfactorily to themselves, to the accused, or to the community; that a separate foundation should be established for the confinement, correction, and cure of those who escape trial or conviction for crimes, whether on false or on well-proved pleas of insanity; or who are of criminal propensities; and who for either of these causes are, or ought to be, the legal subjects of personal restraint; and that a commission of lunacy and idiocy should be appointed, under suitable legal sanctions and responsibilities, for the purposes we have designated. Until these things be done, no reasonable approximation to a complete provision for the real necessities of society and its insane members, on a subject most important to the welfare of both, is to be expected.—*Am. Jour. of Insanity.*

